# Can Comprehensive Geriatric Assessment Predict Tolerance of Radiotherapy for Localized Prostate Cancer in Men Aged 75 Years or Older?

**DOI:** 10.3390/cancers12030635

**Published:** 2020-03-09

**Authors:** Aurore Goineau, Loïc Campion, Jean-Marie Commer, Brigitte Vié, Agnès Ghesquière, Guillaume Béra, Didier Jaffres, Nicolas Magné, Xavier Artignan, Jérôme Chamois, Philippe Bergerot, Gilles Créhange, Elisabeth Deniaud-Alexandre, Xavier Buthaud, Yazid Belkacémi, Mélanie Doré, Laure De Decker, Stéphane Supiot

**Affiliations:** 1 Department of Radiation Oncology, Institut de Cancérologie de l’Ouest, 49000 Angers, France; jean-marie.commer@ico.unicancer.fr; 2 Department of Statistics, Institut de Cancérologie de l’Ouest, CRCINA, University of Nantes, INSERM UMR1232, CNRS-ERL6001, 44800 Saint Herblain, France; loic.campion@ico.unicancer.fr; 3 Department of Radiation Oncology, Clinique Armoricaine de Radiologie, 22000 St Brieuc, France; b.vie@cario-sante.fr (B.V.); aghesquiere@ch-treguier.fr (A.G.); 4 Department of Radiation Oncology, Centre Hospitalier de Bretagne Sud, 56100 Lorient, France; g.bera@ch-bretagne-sud.fr (G.B.); d.jaffres@ch-bretagne-sud.fr (D.J.); 5 Department of Radiation Oncology, Institut de Cancérologie de Loire, 42270 St Priest en Jarez, France; nicolas.magne@icloire.fr; 6 Department of Radiation Oncology, CHP St Grégoire, 35760 St Grégoire, France; xartignan@vivalto-sante.com (X.A.); jchamois@vivalto-sante.com (J.C.); 7 Department of Radiation Oncology, Clinique Mutualiste de l’Estuaire, 44600 St Nazaire, France; philippe.bergerot@mla.fr; 8 Department of Radiation Oncology, Centre Georges François Leclerc, 21000 Dijon, France; gcrehange@cgfl.fr; 9 Department of Radiation Oncology, Centre Hospitalier Départemental de Vendée, 85000 La Roche sur Yon, France; elisabeth.deniaud-alexandre@chd-vendee.fr; 10 Department of Radiation Oncology, Centre Catherine de Sienne, 44000 Nantes, France; xavier.buthaud@groupeconfluent.fr; 11 Department of Radiation Oncology, CHU Henri Mondor, 94000 Créteil, France; yazid.belkacemi@aphp.fr; 12 Department of Radiation Oncology, Institut de Cancérologie de l’Ouest, 44800 Saint Herblain, France; melanie.dore@ico.unicancer.fr (M.D.); laure.dedecker@ico.unicancer.fr (L.D.D.); stephane.supiot@ico.unicancer.fr (S.S.)

**Keywords:** prostate cancer, radiotherapy, quality of life, toxicity, comprehensive geriatric assessment, geriatric rating scales

## Abstract

Curative radiotherapy for prostate cancer is common in the elderly. However, concerns about potential toxicity have inhibited access to radiotherapy for this population, for whom preserving quality of life (QoL) is crucial. The primary endpoint was to identify predictors of impaired QoL in men aged 75 years or older treated with curative intent radiotherapy with or without androgen deprivation therapy (ADT) for localized prostate cancer. We prospectively performed comprehensive geriatric assessment (CGA) and administered QoL questionnaires to 208 elderly (>75 years) patients prior to, plus two and six months after, radiotherapy (NCT 02876237). The median age of the patients was 77 years (range 75–89). At the start of the study, comorbidities were highlighted in 65% of patients: 23% were depressed, 23% had cognitive impairment, and 16% had reduced independence. At six months, 9% of patients had a consistently decreased QoL (>20 points), and a further 16% had a more moderate reduction (10 to 20 points) in QoL. None of the parameters studied (tumor characteristic, treatment, or oncogeriatric parameters) were predictive of a reduced QoL following radiotherapy. Though co-existing geriatric impairment was common, QoL was maintained for 75% of patients six months after radiotherapy. CGA was poorly predictive of tolerance of prostatic radiotherapy. Geriatric assessments dedicated to quality of life following radiotherapy need to be developed.

## 1. Introduction

Incidence of prostate cancer is on the increase in Western countries, especially in the elderly, and determining the best treatment to offer this heterogeneous population is a challenging issue. Quality of life (QoL) is key in this group of patients, and the potential toxicity of radiotherapy is a major concern, both for patients and the physicians advising them. Moreover, comorbidities or impaired urinary, digestive, or geriatric functions are more frequent in the elderly and may worsen after radiotherapy. Palliative treatment (androgen deprivation therapy (ADT) or no therapy at all) is thus offered to most patients over the age of 75 years with localized high-risk prostate cancer [1], despite the proven improvement in survival induced by radiotherapy [2].

We chose to evaluate early quality of life after radiotherapy because we have previously shown, in a younger population, that there is a temporary decrease in QoL after intensity-modulated radiation therapy (IMRT) for prostate cancer [3], but that most symptoms resolve within 6 months and long-term quality of life is usually similar to baseline [4]. Good tolerance of prostate cancer radiotherapy in the elderly has been reported in retrospective studies [5], but there is a lack of prospective data.

Adapting oncological treatment to the profile of elderly patients remains a challenge. Geriatric screening tools have been developed to orient frail patients before any oncologic intervention [6], but comprehensive geriatric assessment (CGA) provides a more accurate evaluation. Although CGA is common prior to surgery, chemotherapy [7], or ADT [8], specific studies on prostate cancer radiotherapy in elderly patients have mostly been retrospective, lacking in accurate geriatric evaluation [9], or have focused on toxicity (physician-reported) rather than quality of life (self-reported) [10]. Our study was designed to address this gap and determine whether using CGA is capable of predicting QoL outcomes following radiotherapy for localized prostate cancer in the elderly. 

## 2. Results

The study included 230 patients from 11 different cancer centers. Twenty-two patients were excluded from the analysis: One patient was too young (73 years), and quality of life questionnaire were missing for ten patients at the baseline, and for eleven at both two and six months (supplementary material Figure S1). In total, 208 patients were analyzed (Table 1). According to D’Amico’s classification, most presented with intermediate- (42.7%) or high-risk (48.6%) prostate cancer; 47.6% received concomitant ADT. The median age was 77 years (from 75 to 89). Patients traveled a median of 23 km (mean 30 km; range 2–147) from their homes to the radiotherapy center. 

### 2.1. Urinary and Geriatric Impairment Over Time

Before radiotherapy, 2.5% of patients (5/200) suffered from severely (International Prostate Symptom Score (IPSS) 20–35) impaired urinary function, and 4.4% (8/182) did at 6 months. Function was moderately impaired (IPSS 8–19) in 38.5% (77/200) of patients before radiotherapy and in 37.9% (69/182) at six months (Table 2).

The Cumulative Illness Rating Scale for Geriatrics (CIRS-G) revealed some degree of comorbidity in 64.5% of patients; at least one severe comorbidity (i.e., at least one organ system with grade 3–4 comorbidity) was present in 28.2% of patients at baseline and in 64.9% at 6 months. A Geriatric Depression Scale (GDS) score ≥ 1 highlighted depressive symptoms in 48 patients (23%) before treatment, and 47 patients (22.6%) at six months. Before treatment, the activities of daily living (ADL score > 6) and the instrumental activities (Instrumental Activities of Daily Living (IADL) > 7) were compromised in 26 (12.5%) and 34 (16.3%) patients, respectively. At 6 months, they were impaired in 26 (12.5%) and 32 (11.1%) patients, respectively. Forty-eight (23.1%) patients presented cognitive disorders (Mini Mental State Examination (MMSE) < 27) before treatment and 53 (25.5%) after 6 months. The assessed risk of fall (Get Up And Go Test (GUAGT) < 0) was 8.2% before and 7.7% after radiotherapy, and undernutrition (Mini Nutritional Assessment (MNA) ≤ 17) was found in one patient.

### 2.2. Evolution in Quality of Life

**Figure 1:**
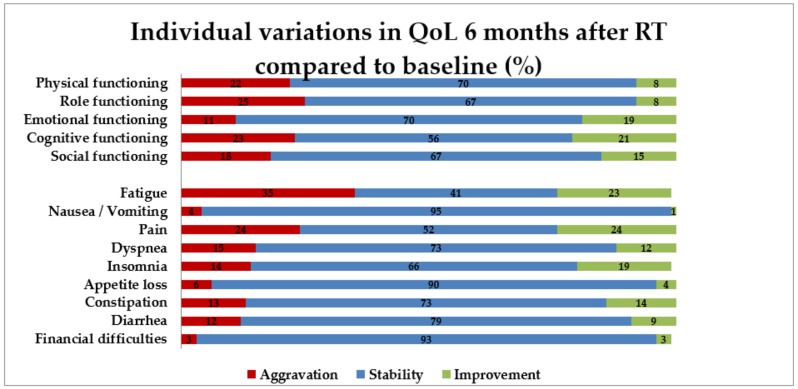
Individual variations in quality of life six months after radiotherapy compared to baseline. QoL: quality of life; RT: radiotherapy.

Patient-reported QoL following radiotherapy is shown in Figure 1 (individual data). For 182 patients, the QLQ C30 questionnaire was available before treatment and at 6 months. According to this questionnaire, at 6 months, a moderate decrease in general QoL (loss of 10 to 20 points) was found in 29 patients (16%) and a marked one (≥ 20 points) in another 16 patients (8.8%). The most frequent QoL alterations were fatigue (64 patients), role functioning (46), pain (43), cognitive functioning (41), and physical functioning (40).

**Figure 2:**
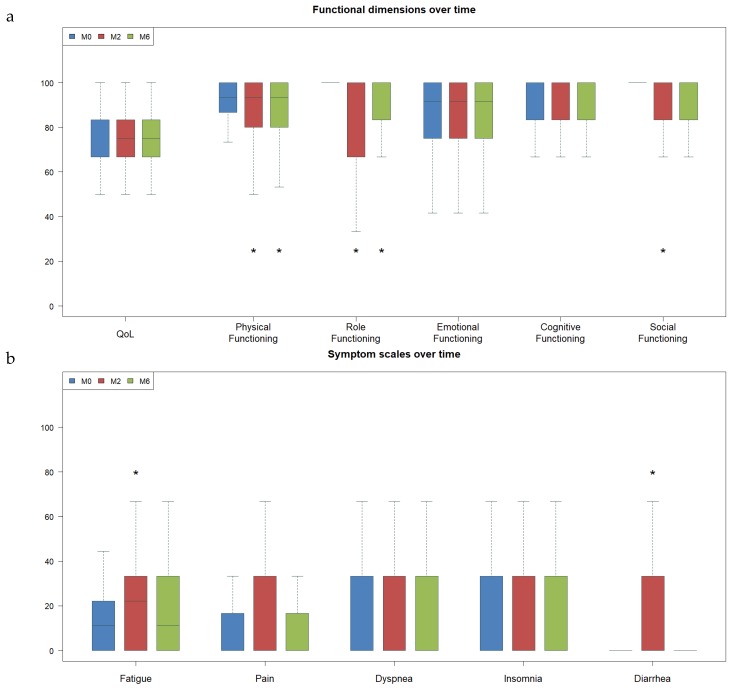
Quality of life over time (means comparison). For each item in the QLQ C30 questionnaire, we compared mean scores before, and two and six months after, radiotherapy. For the functional scales (**a**), higher scores represented better QoL. For the symptom scores (**b**), higher scores represented more severe symptoms and worse QoL. *Statistically significant variations (*p* < 0.05). For symptom scales, items without any variations (nausea, appetite loss, constipation, and financial difficulties) have not been represented to make the figure easier to read.

Figure 2 shows the evolution over time of each item on the QLQ C30 questionnaire (mean comparisons between baseline, and two and six months). At two months, we found a significant (*p* < 0.01) decrease in physical (mean loss −3, *p* = 0.0001), role (mean loss −9, *p* < 0.0001), and social (mean loss −3, *p* = 0.007) functioning. For symptoms, changes in fatigue (mean increase +7, *p* < 0.0001) and diarrhea (mean increase +6, *p* = 0.003) were significant at two months. Only physical and role functioning remained significantly impaired at six months (mean loss −3 and −7, *p* = 0.0001 and *p* < 0.0001, respectively).

### 2.3. Predictive Factors for Qol Impairment

The aim of our study was to use initial clinical or geriatric settings to identify the patients at risk of QoL impairment after prostate cancer radiotherapy. Unfortunately, we found no external parameter capable of predicting a significant decrease in general QoL. Only QoL deterioration at two months was correlated with worsening QoL six months after prostate cancer radiotherapy (*p* = 0.023) (Table 3).

## 3. Discussion

Identifying which elderly patients will benefit from local treatment for prostate cancer is still a challenge, and prospective studies on QoL after radiotherapy in this population were lacking. Our exhaustive and systematic documentation of CGA before and six months after treatment, has, to the best of our knowledge, created a unique dataset.

The purpose of this study was to collect a significant amount of data (relating to the patients, their tumors and specific geriatric characteristics) and to determine which could predict a decrease in QoL after radiotherapy. However, neither initial geriatric assessment items, oncologic data, distance to radiotherapy center, nor adding a concomitant ADT to radiotherapy could be associated with impaired QoL at six months. This may be explained by several factors.

First, the initial CGA may have identified impairments, and in turn, corrective measures, that may have contributed to improved tolerance. This potential bias was identified during the study design process, but to failure to implement these corrective measures once the impairment had been identified would have been unethical. Secondly, the number of patients in our study who experienced decreased QoL was limited. One study that reported higher levels of comorbidity did find that comorbidity was predictive of impaired long-term QoL after prostate cancer radiotherapy associated with ADT [11]; another, like this study, found no CGA parameter that was predictive for significant radiotherapy toxicity [10]. It is possible that the G8 and CGA tools are poorly-adapted to radiotherapy and localized cancer [12], having been principally developed for chemotherapy or major surgery [13,14]. Several studies have emphasized the determinativeness of nutritional status before treatment on the smooth running of chemotherapy, and even on survival [13,15]. That is why, on the G8 (validated geriatric screening questionnaire), nutrition assessment has prominence (three questions out of eight). On the other hand, undernutrition was practically absent from our study. Similarly, geriatricians usually give importance to the risk of falls as a mark of fragility [13] but fewer than 10% of our patients were found to be at high risk of falling. This suggested that screening tools such as the G8 seem ill-suited to detecting vulnerabilities in this particular population, but even with the fuller CGA used in our study, we failed to predict tolerance of radiotherapy [10]. The development and evaluation of new tools are needed.

Despite many geriatric frailties and several comorbidities before treatment, 75% of our patients had maintained or improved their overall QoL at study completion. As our study population seemed representative of the routine clinical elderly prostate cancer population, we suggest that earlier fears of radiotherapy toxicity in this group may have been overemphasized. Our baseline CGA showed that most patients had numerous fragilities. Two thirds of the patients in our study presented with comorbidities, sometimes severe (28.2%), which may affect the tolerance of ADT. Unsuspected cognitive disorders and depression were highlighted in nearly 20% of patients, which may also be aggravated by ADT [16]. In addition, one in six patients suffered from a loss of autonomy. 

Transient acute toxicity was observed in certain patients. Severe urinary symptoms were present in 2.5% of patients at the baseline, compared with 4.4% at 6 months, with a peak of 7.5% at 2 months. Previous studies have found that late urinary toxicity is more frequent in elderly patients (>70) [17,18]. Similarly, diarrhea was significantly increased at 2 months but returned to the baseline level at 6 months in our study. Other authors have shown that older patients are also at higher risk of digestive toxicity [19,20]. Radiotherapy also temporarily increased fatigue, with possible consequences for social and leisure interactions. There are many potential causes of asthenia during radiotherapy: ADT, nocturia, comorbidities, or trips to the cancer center may all contribute. Fatigue is frequent in men undergoing radiotherapy for prostate cancer, especially among those treated with concomitant ADT [21,22], but whether this symptom is more pronounced in elderly patients remains debated. 

Despite acute toxicity, overall QoL was maintained or improved in 71% of patients at 2 months and 75% at 6 months. Only 8.8% of patients had severely decreased QoL at six months (> 20 points). Our good results are consistent with data found in younger patients, in whom short-term ADT associated with radiation therapy did not greatly impair physical or mental health in comparison with active monitoring [23]. We previously published a longitudinal evaluation of QoL after intensity-modulated prostate radiotherapy in younger subjects (age range 50–80 years, median age 73) and found consistent results: quality of life at six months was generally similar to the baseline after temporary impairment at two months [3,4]. Other authors have also reported a moderate but transient impaired QoL after radiotherapy in younger patients [24]. Although a direct comparison has not been scientifically validated, we note that 25% of patients in this geriatric series experienced a 6-month QoL decrease versus 15.8% in our series with younger patients. The impact of age on the tolerability of radiotherapy remains an open question.

Our uniquely exhaustive CGA before and six months after treatment found that geriatric parameters appeared stable over time, except for cognitive impairment. This suggests that prostate cancer radiotherapy does not reduce patient autonomy or mood, which are major elements in quality of life and independent daily living in the elderly. Cognitive impairment, present in 23% of patients before treatment and 25.5% after 6 months, may be related to ADT [25] but this remains open for debate [26].

Too many patients are currently offered only ADT, having been judged arbitrarily as too old or frail for radiotherapy, without rigorous geriatric evaluation [27]. We have shown that QoL is mainly preserved or improved after prostate cancer radiotherapy, even in the presence of impaired geriatric parameters, and believe that patients should be offered the most effective oncological treatment based on geriatric evaluation.

Our study may have some limitations. First, we tried to minimize the selection bias through the multicentric nature of this study, not limited to teaching hospitals. Beyond the age of 75 years, radical prostatectomy is not recommended because of the higher risk of urinary toxicity than in younger patients [28]. In our study, only 23 patients (11.1%) were treated with post-operative radiotherapy. It is conceivable that this operated subpopulation may have lower comorbidities as it had previously been considered eligible for prostatectomy. However, in the majority of cases, prostatectomy was performed several years earlier with a biochemical relapse that occurred after the age of 75 years. Post-operative versus exclusive radiotherapy was not predictive for a different quality of life after treatment in our study. In addition, the high number of patients with frailties according to the initial CGA was indicative of the reasonably representative nature of our population. 

Second, this study focused on acute toxicity and early evaluation of QoL. In geriatric populations, with shorter life expectancy than younger patients, the rapid restoration of quality of life seemed all the more important to us. Furthermore, we have previously shown that QoL, after temporarily worsening, returned to the baseline between 6 and 18 months after the end of radiotherapy. However, our results need to be confirmed after a longer follow-up period, knowing that digestive toxicities can appear several years after the end of the treatment [23]. 

The great originality of our study is its extensive and prospective geriatric evaluation, carried out prior to treatment and renewed at 6 months.

## 4. Patients and Methods

The inclusion criteria for our prospective, multicenter cohort study (NCT 02876237) were as follows: men aged 75 or more, with localized prostate cancer, and a proposal for radiation therapy (multidisciplinary tumor board) with curative intent, either alone or combined with ADT. The appropriate French ethics committees approved the study (number 2014-A00300-47), and all patients gave consent for participation. A detailed description of the study methods has been published previously [29]. 

In this study, we assessed QoL at 2 months because this period corresponds to acute side effects. We selected a later time point because acute side effects resolved within 6 months in our previous study [4].

The patients completed 3 questionnaires before radiotherapy, and at two and six months after treatment: the International Prostate Symptom Score (IPSS), the International Index of Erectile Function (IIEF-5), and the European Organization for Research and Treatment of Cancer (EORTC) quality of life (QLQ C30 version 3.0). Items were combined according to EORTC criteria into several scales ranging from 1 to 100. Higher scores for global health and function indicate better quality of life (QoL); higher symptom scores indicate poorer QoL. A geriatrician performed a comprehensive geriatric assessment prior to radiotherapy and six months after. This CGA included current medication, body mass index, home to study center distance, the Cumulative Illness Rating Scale for Geriatrics (CIRS-G), activities of daily life (ADL), instrumental activities of daily life (IADL), Mini-Mental State Examination (MMSE), Mini Geriatric Depression Scale (GDS), Mini Nutritional Assessment (MNA), and the Get Up And Go Test (GUAGT). 

The primary endpoint was to identify the predictive factors for impaired QoL after radiotherapy, defined as a decrease in overall QLC30 score of more than 10 points at 2 or 6 months compared with baseline. Secondary endpoints were to report toxicity (urinary and sexual functions) and evolution in geriatric parameters (CGA) over time.

Predictive factors for QoL variations (impact of tumor characteristics, treatment, or CGA parameters) were screened for using the Fisher test (categoric variables) or the Mann and Whitney test (continuous variables). The Wilcoxon test was used to analyze evolution in QoL parameters from the baseline. The significance threshold was *p* < 0.05, except for changes in QoL over time (*p* < 0.01). A clinically relevant change in QoL was defined as a >10-point change [30,31]. 

We expected approximately 25%–30% of patients to have a >10-point decrease in overall QoL (29–30 items of QLQ C30 scale). We chose the Severity Index Score (from CIRS-G, which evaluates comorbidities) as a representative geriatric scale because in current daily clinical practice, in elderly patients, comorbidities seem to be the main criterion of choice between palliative (ADT) and curative treatment (radiotherapy). We expected about 25% of patients to be classified with a high Severity Index Score (>2).

We expected that patients with a Severity Index Score of >2 would more often have a >10-point decrease in QoL (40% vs. 20%, OR = 2.67).

In order to dispose of at least 80% power to detect such an OR value with alpha risk = 5% (one-sided), we had to include at least 184 evaluable patients.

## 5. Conclusions

We did not find any predictors of declining quality of life following radiotherapy. Despite numerous comorbidities and geriatric fragilities in our population, QoL mostly appeared to be maintained or improved after radiotherapy, with or without ADT. These data suggest that the fear of an iatrogenic decrease in QoL should not result in abandoning a curative treatment that is likely to improve the life expectancy of these patients. Moreover, performing an extensive CGA prior to treatment can reveal unsuspected frailties that can worsen during or after treatment, but also and more importantly provides an opportunity for them to be managed appropriately. Further evaluation of long-term QoL and geriatric assessments to refine the prediction of tolerance of radiotherapy are needed.

## Figures and Tables

**Table 1:** Patient characteristics (N = 208).

Characteristics	Characteristics	N (%)
Age	75–79	154 (74.0)
	80–84	49 (23.6)
	≥85	5 (2.4)
BMI	underweight (<18)	0 (0.0)
	normal (18–25)	71 (34.6)
	overweight (25–30)	94 (45.9)
	obesity (>30)	40 (19.5)
Number of medications	0–3	107 (51.4)
	>3	109 (48.6)
Distance to radiotherapy center	<30 km	119 (57.2)
	30 to 60 km	57 (27.4)
	≥60 km	32 (15.4)
Clinical stage	low	18 (8.7)
	Intermediate	88 (42.7)
	high	100 (48.6)
Radiotherapy	prostate	185 (88.9)
	prostate bed	23 (11.1)
ADT	yes	99 (47.6)
	no	109 (52.4)

BMI: body mass index; ADL: activities of daily living; IADL: instrumental activities of daily living; ADT: androgen deprivation therapy.

**Table 2:** Urinary and geriatric impairments over time.

**Geriatric problems**	**M0 N = 208 (%)**	**M6 N = 208 (%)**
Depression	48 (23)	47 (22.6)
Impaired GUAGT	17 (8.2)	16 (7.7)
Malnutrition	1 (0.5)	1 (0.5)
Comorbidities	134 (64.5)	135 (64.9)
ADL impairment	26 (12.5)	26 (12.5)
IADL impairment	34 (16.3)	32 (11.1)
Cognitive impairment	48 (23)	53 (25.5)
**Urinary function (IPSS score)**	**M0 N = 200 (%)**	**M6 N = 182 (%)**
Severely impaired (IPSS 20–35)	5 (2.5)	8 (4.4)
Moderately impaired (IPSS 8–19)	77 (38.5)	69 (37.9)
Mildly impaired or normal function (IPSS <8)	118 (59)	105 (57.7)

GUAGT: Get Up And Go Test; ADL: activities of daily living; IADL: instrumental activities of daily living; IPSS: International Prostate Symptom Score; M0: study entry, before radiotherapy; M6: six months after radiotherapy.

**Table 3:** Predictive factors for changes ≥ 10 points in quality of life scores at M6/M0.

Predictive Factors	Predictive Factors	No QoL Decrease (n = 136)	QoL Decrease (n = 45)	*p*
Age (+/- SD)		78.1 (+/- 2.5)	78.2 (+/- 2.9)	0.779
Distance (+/- SD)		29.9 (+/- 24.9)	31.9 (+/- 23.7)	0.638
BMI (+/- SD)		26.6 (+/- 3.9)	27.9 (+/- 5.5)	0.098
Number of medications (+/- SD)		3.3 (+/- 2.6)	2.9 (+/- 2.2)	0.429
QoL decrease at M2	<10 ≥10	95 30	21 17	0.023
Clinical stage at M0	Low Intermediate High	13 64 57	2 16 27	0.114
Radiotherapy	prostate prostatic bed pelvis	102 13 21	36 7 2	0.106
ADT	No Yes	78 58	20 25	0.167
Depression at M0	No Yes	106 30	35 10	0.982
Risk of fall at M0	No Yes	108 9	38 5	0.528
Malnutrition at M0	No Yes	135 1	45 0	0.999
Comorbidities at M0	No: <4 Yes:4+	32 88	16 27	0.193
Comorbidities gr3–4 at M0	0 1 or more	85 35	32 11	0.654
Urinary symptoms at M0	Light Moderate High	74 51 5	29 15 0	0.368
Urinary symptoms at M2	Mild Moderate Severe	54 60 11	19 15 1	0.367
ADL impairments at M0	No Yes	119 16	39 6	0.793
IADL impairments	No Yes	117 18	36 9	0.278
Cognitive impairment	No Yes	108 28	34 11	0.585

BMI: body mass index; QoL: quality of life; M0: study entry, before radiotherapy; M2/M6: two/six months after radiotherapy; ADT: androgen deprivation therapy; ADL: activities of daily living; IADL: instrumental activities of daily living.

## Supplementary Materials

The following are available online at https://www.mdpi.com/2072-6694/12/3/635/s1, Figure S1: Flow chart.
