# Supplementary figures and images for: Can Comprehensive Geriatric Assessment Predict Tolerance of Radiotherapy for Localized Prostate Cancer in Men Aged 75 Years or Older?
Source: Cancers (Basel). 2020 Mar 9;12(3):635. doi: 10.3390/cancers12030635 (PMC7139355; doi:10.3390/cancers12030635)

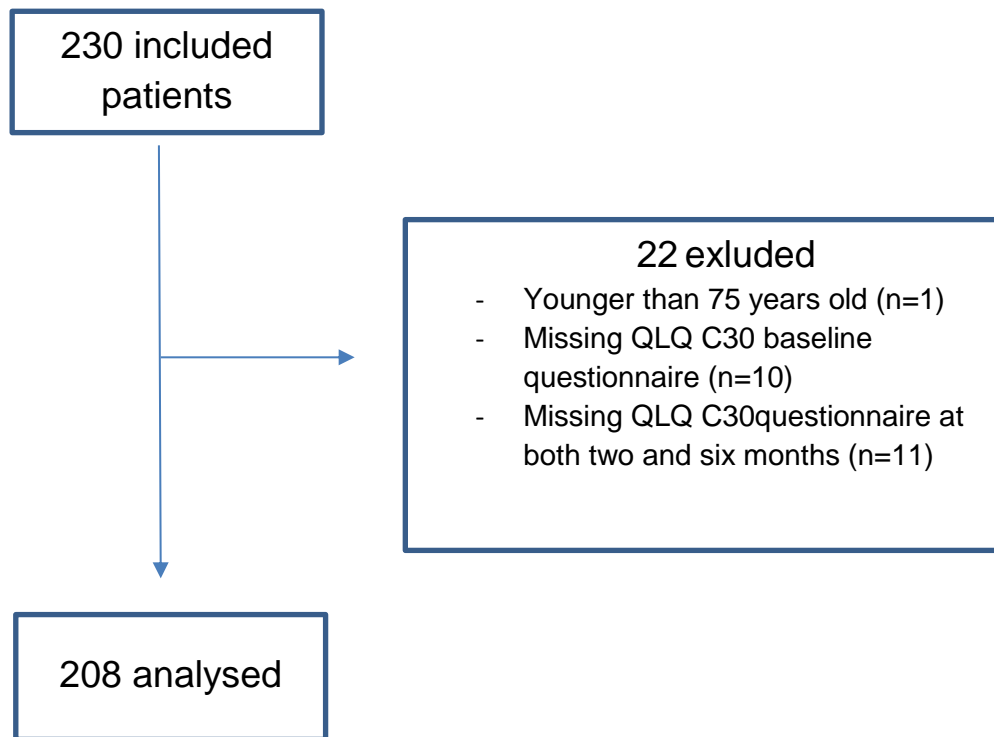

Supplement: Supplementary file 1 [file cancers-12-00635-s001.pdf]
